# Clinical practice considerations for restarting pegvaliase in adults with phenylketonuria

**DOI:** 10.1016/j.ymgmr.2026.101333

**Published:** 2026-08-10

**Authors:** Markey McNutt, Katherine J. Anderson, Ashley Andrews, Erin Cooney, Brittany M. Murray, Michelle Tharp, Erika Vucko, Leah Wessenberg, Kassi Wilson, Derek Wong, Joyanna Hansen, Sarah Rose, Bridget Wardley, Janet A. Thomas

**Affiliations:** a UT Southwestern Medical Center, Dallas, TX, USA; b University of Vermont Health, Burlington, VT, USA; c University of Utah, Salt Lake City, UT, USA; d University of Texas Medical Branch, Galveston, TX, USA; e Boston Children's Hospital, Boston, MA, USA; f University of Mississippi Medical Center, Jackson, MS, USA; g Ann & Robert H. Lurie Children's Hospital, Chicago, IL, USA; h Oregon Health and Science University, Portland, OR, USA; i University of South Florida, Tampa, FL, USA; j David Geffen School of Medicine at UCLA, Los Angeles, CA, USA; k BioMarin Pharmaceutical Inc., Novato, CA, USA; l University of Colorado School of Medicine and Children's Hospital Colorado, Aurora, CO, USA

**Keywords:** Phenylketonuria, PKU, Phenylalanine, Pegvaliase, Restart, Case series, Best practices

## Abstract

**Objective:**

To provide key considerations and best practices for restarting pegvaliase based on real-world experiences of healthcare professionals managing individuals with phenylketonuria (PKU).

**Methods:**

An in-person advisory board with 11 experienced PKU practitioners identified strategies for restarting pegvaliase after a treatment pause. The advisors presented real-world restart cases detailing treatment history, reasons for discontinuation, restart approach, and outcomes, followed by discussions about best practices for clinical decision-making.

**Results:**

Among 11 restart cases, reasons for treatment discontinuation included adverse events (AEs) (3 cases), limited blood phenylalanine (Phe) response (4 cases), clinical trial participation (1 case), and pregnancy (3 cases). Time off treatment ranged from 3 to 64 months. Eight cases restarted pegvaliase with an expedited titration schedule, while 2 cases resumed with a slowed approach and 1 case used the standard titration. At the time of the advisory board, 9 cases had achieved blood Phe ≤360 μmol/L, 1 case still had elevated blood Phe levels but had not yet completed titration and 1 case discontinued after 18 months on therapy.

Key considerations for restarting pegvaliase after AE-related discontinuations include proactively addressing anxiety, optimizing premedications, ensuring access to on-demand medications and using a flexible, individualized titration approach. For individuals discontinuing primarily due to a limited blood Phe response, resuming at the previously tolerated dose and titrating adaptively as tolerated may be considered. For individuals desiring an expedited titration, more frequent blood Phe monitoring may be warranted to guide dietary adjustments and dosing. Life changes, individual preferences and social determinants of health should inform timing and resources for restart. For pregnancy-related discontinuations restart decisions should be made collaboratively with the individual and care team (geneticist, metabolic dietitian, obstetrician, and other relevant caregivers).

**Conclusion:**

Pegvaliase can help achieve blood Phe control, which may mitigate neurocognitive effects and can result in greater dietary flexibility and improved quality of life. Restarting pegvaliase is feasible for most individuals, with many having a better experience during the restart process compared with the first treatment initiation. AEs were reported (eg, injection site reactions, rash, and arthralgia) but were milder compared to the previous treatment course in most cases and some individuals responded at lower doses or after shorter treatment duration. Restarting pegvaliase should be considered as part of a shared decision-making process for individuals seeking to further optimize outcomes.

## 1 Introduction

Phenylketonuria (PKU), also known as phenylalanine hydroxylase (PAH) deficiency, is an inherited metabolic condition characterized by the inability to effectively convert phenylalanine (Phe) to tyrosine, leading to elevated Phe levels in the blood and brain [1]. The American College of Medical Genetics and Genomics (ACMG) management guidelines recommend that individuals with PKU are treated for life, with evidence indicating that maintaining blood Phe levels below 360 μmol/L is associated with optimal developmental and neurocognitive outcomes [2], [3], [4], [5].

Traditional management is challenging and, particularly in adulthood, largely suboptimal. Historically, PKU treatment relied solely on medical nutrition therapy to reduce Phe intake by following a diet very restricted in natural protein (limited to 5–7 g per day in individuals with classical PKU) and supplemented with a specialized metabolic formula (medical food) and low-protein or protein-modified foods to meet protein, caloric, and micronutrient needs [6]. Sapropterin, a synthetic analogue of tetrahydrobiopterin (BH4, the cofactor for PAH) and more recently sepiapterin, a BH4 precursor, are approved to lower blood Phe in responsive individuals in conjunction with a Phe-restricted diet [7], [8]. The chronic nature of PKU warrants lifelong management, placing a burden on individuals with PKU and affecting quality of life [9], [10], [11]. Many individuals with PKU have lapses in treatment or periods of being “lost to follow-up” in which they may have an extended period of time that they do not present to clinic for PKU care. Struggles with adherence start to become more pronounced during adolescence and often extend into adulthood [12], [13].

Pegvaliase, a bacterially-derived enzyme substitution therapy administered via subcutaneous injection, offers a paradigm shift from historical treatment options. In many cases, treatment with pegvaliase can lower blood Phe without requiring a Phe-restricted diet [14]. In Phase 3 clinical trials pegvaliase demonstrated substantial and sustained reductions in blood Phe in the majority of persistent users, while also allowing for increased intake of natural protein [6], [15], [16]. In a subset of 250 trial participants administering pegvaliase, 63.3% were no longer consuming medical formula at their last diet assessment [6].

Pegvaliase is administered using a gradual induction, titration, and maintenance (I/T/M) regimen allowing dose escalation based on individual tolerability of adverse events (AEs) and treatment response. Once efficacy is achieved, the treatment can be tailored to the lowest effective and tolerated dose. As a bacterially-derived enzyme, pegvaliase stimulates an immune response, which is partially mitigated by the I/T/M dosing regimen, and matures over time. Despite this, hypersensitivity events including injection site reactions, rash, and arthralgia are common, especially during early treatment. Most AEs are mild to moderate, self-limited, and manageable with pre-dose H1/H2 receptor antagonists to lessen or prevent hypersensitivity reactions, antipyretics and non-steroidal anti-inflammatory drugs as needed. Acute systemic hypersensitivity reactions, including non-IgE-mediated anaphylaxis, occur less frequently but require immediate medical intervention [15], [16].

Time to reach efficacy with pegvaliase is variable (median [Q1, Q3] time to ≤360 μmol/L in phase 3 trials was 10.0 [6.0, 16.0] months), and intolerable AEs may prolong the time to achieve an efficacious response [17]. This delay in blood Phe reduction, coupled with treatment/injection fatigue, can result in discontinuation of therapy. Furthermore, life events such as loss of insurance, changes in employment, relocation and family planning can also impact an individual's decision to discontinue treatment. Nevertheless, restarting pegvaliase following a treatment interruption is possible. During the phase 3 clinical trials, 28 individuals underwent an 8-week treatment break during the randomized discontinuation period. Following the blinded placebo phase, these individuals successfully resumed pegvaliase at their previously randomized maintenance dose [18].

Since approval by the Food and Drug Administration (FDA) in 2018, there is growing experience with restarting pegvaliase therapy in clinical practice, and providers and individuals with PKU may benefit from these shared learnings. Herein, we report on real-world experiences of 11 individuals with PKU who resumed pegvaliase therapy after treatment discontinuation, along with key considerations for approaching a restart based on the initial treatment course.

## 2 Methods

An in-person advisory board meeting was held in November 2024, bringing together 11 experienced PKU practitioners (medical geneticists and nurse practitioners) from the United States to discuss strategies for restarting pegvaliase after treatment discontinuation. Clinic-level data from individuals with PKU who had resumed pegvaliase therapy following a pause of at least 3 months were collected and aggregated to provide an overview of the restart experiences.

During the meeting, each advisor presented 1 of these restart cases in more detail, sharing information on relevant medical history, treatment course and duration prior to discontinuation (such as blood Phe levels and AEs), dose at last exposure, reasons for discontinuation, duration of treatment pause, dose at restart, restart approach and the individual's treatment status at the time of the advisory board. Following each case presentation, a targeted discussion allowed for sharing of best practices to support clinical decision-making.

All individuals provided voluntary consent for their de-identified information to be presented as a case at the advisory board presentations and for inclusion in this publication.

## 3 Results

### 3.1 Consolidated clinic restart experience

In total, the clinics represented by the 11 advisors had restarted 30 individuals after a pause in pegvaliase treatment. Among these 30 individuals, the median time on therapy prior to discontinuation was 13.5 months (range 0–120 months) and the median duration of treatment pause was 20.5 months (range 3–132 months). The most common dose administered upon restart was 2.5 mg once or twice weekly, although some individuals resumed therapy at higher doses. At the time of the advisory board, 76.7% (23/30) of individuals continued pegvaliase treatment.

### 3.2 Presented restart cases

#### 3.2.1 Clinical case characteristics

Table 1 summarizes the treatment course of the 11 cases presented during the advisory board; a detailed narrative is provided for each case in the supplement. Overall, 3 cases had previously discontinued due to AEs, 4 due to limited blood Phe response, 1 due to participation in a clinical trial and 3 due to pregnancy. Two cases were off treatment for 3–6 months, 2 for 6–12 months, 2 for 1–2 years and 5 for >2 years. Eight cases were able to restart pegvaliase with an expedited titration schedule, while 2 cases resumed with a slowed approach and 1 case used a standard scheme (Table 2). At the time of the advisory board, the majority (9 cases) had achieved blood Phe levels below 360 μmol/L, while one case was still in the titration process with blood Phe levels exceeding 360 μmol/L and 1 discontinuation had occurred.

**Table 1:** Demographics and treatment journeys of 11 presented cases resuming pegvaliase therapy.

Case	1	2	3	4	5	6	7	8	9	10	11
**Demographics & background**											

Age (current), years	45	30	50	24	25	40	37	37	28	42	29

Sex	M	F	F	M	M	F	M	F	F	F	F

Notable Medical History	Anxiety, Depression, Delayed PKU Dx (17mo)	Asthma, POTS	Anxiety, Depression, GERD	N/A	Eosinophilic esophagitis	Obesity, s/p gastric sleeve	Anxiety, ADHD, Depression	N/A	N/A	Anxiety w/ panic attacks, Depression	N/A

**Prior course of treatment**											

Initiated in Clinical Trial	N	N	Y	N	N	Y^a^	Y	Y	N	Y	N

Baseline Phe, μmol/L	407	1058	1614	875	1378	1155	2052	~600	1440	~1200	1636

Length of treatment, months	~3	38	6	37	20	20	59	123	27	76^b^	23

Notable Adverse Events (AEs)	Recurrent leg rash	ASHR	Severe abdominal pain, N/V with injection	ASHR, severe joint pain	Joint pain, chills, ISRs	Mild ISRs, large softball size knot (ISR)	None reported	Rash, hives, severe joint pain	Mild headache, ISR, joint pain, indigestion	ISRs, lymphadeno-pathy, joint pain, hives	Alopecia

Notable life/treatment events	Referral for leg rash	Single parent	Emergency room visit for AEs	Moved to college, Crohn's diagnosis	Difficulty w/ injections/lab draws	Hesitant to dose increase	Participant in Phase 1 & 2 studies	Liberalized diet, Phe 30–120 μmol/L ~ 10 years	Pregnancy, remained on pegvaliase	~9 mo break in pegvaliase therapy for pregnancy	Marriage

Achieved Phe ≤ 360 μmol/L	UNK	Y	N	Y	N	N	Y	Y	Y	Y	Y

Last Phe prior to D/C, μmol/L	UNK	774	UNK	667	814	768	2071	2	217	UNK	434

Dose at D/C	20 mg QD	10 mg QD	40 mg QD	60 mg QD	60 mg QD	20 mg QD	4.5 mg/kg/wk^c^	20 mg, 3×/wk	20 mg QD	UNK	10 & 20 mg, alt. daily

Reason for D/C	AE	AEs, COVID-19	AEs	Phe variability	Limited blood Phe response, interest in clinical trial	COVID-19, hesitant re: 40 mg QD	Missed trial visits, withdrawn	Enrolling in clinical trial	Newborn, sporadic dosing	Planned pregnancy (#2)	Planned pregnancy

**Discontinuation period & reason for restart**											

Months off pegvaliase	8	21	61	4	30	30	64	18	3	10	25

Notable aspects of time off therapy & reason for restart	Moved to new clinic	New spouse, increased symptoms of high Phe	Off diet, lack of insurance coverage for metabolic formula	Felt worse, encouraged by positive experience of others	Worsened mood, anxiety, decreased energy	Weight loss, desire to reach goal weight with liberalized diet	Hospitalized d/t anxiety, depression, irritability	Could not maintain Phe ≤120 mol/L with desired diet	Increased anxiety, depression	Difficulty at work/tasks, anxiety/ depression, brain fog	High Phe during pregnancy, mPKU

**Restart course**											

Baseline Phe, μmol/L	672	1118	1584	866	1526	486	1609	276	1357	868	1474

Restart dose	2.5 mg weekly	2.5 mg, 3×/wk	2.5 mg weekly	10 mg weekly	2.5 mg weekly	2.5 mg weekly	2.5 mg, 2×/wk	2.5 mg, 2×/wk	20 mg, 5×/wk	10 mg, 4×/wk	2.5 mg, 2×/wk

Titration approach	Slowed	Expedited	Standard	Expedited	Expedited	Slowed	Expedited	Expedited	Expedited	Expedited	Expedited

Length of treatment, months	38	12	18	30+	15	2	73	4	31	39	17

Notable AEs	Elevated bilirubin, hypoPhe	Hives, swelling of hand	ISRs mild, other health concerns	Hives	Fewer AEs, mild joint pain	Mild ISRs	Mild joint pain	Mild hives, ISRs	None reported	Fewer AEs, hives	Night sweats

Notable life/treatment events	Job promotion	Spousal support	Loss of insurance	Varied protein intake	Less frequent Phe testing	N/A	Partnered with LCSW re: SDOH	Got married, resumed unrestricted diet	Inconsistent dosing, LTFU	LTFU ~3 months, off therapy	Pregnancy

Achieved Phe ≤360 μmol/L	Y	Y	N	Y	Y	N^d^	Y	Y	Y	Y	Y

Status at time of advisory board & dose	Continues, 10 mg QOD	Continues, 20 mg QD	D/C, 40 mg QD	Continues, 60 mg QD	Continues, 60 mg QD	Continues with titration	Continues, 80 mg QD	Continues, 10 mg, 4×/wk	Continues, 30 mg, 2d alt. 20 mg, 1 day	Continues, 60 mg QD	Continues, 10 mg & 20 mg, alt. QD

ADHD: Attention Deficit Hyperactivity Disorder, ASHR: Acute Systemic Hypersensitivity Reaction, COVID-19: coronavirus disease 2019, D/C: Discontinuation, GERD: Gastroesophageal reflux disease, hypoPhe: hypophenylalaninemia, ISR: Injection Site Reaction, LCSW: Licensed Clinical Social Worker, LTFU: Lost to follow-up, mo: months, mPKU: maternal PKU, N/V: Nausea & Vomiting, Phe: phenylalanine, POTS: Postural Orthostatic Tachycardia Syndrome, QD: daily, QOD: every other day, SDOH: Social Determinants of Health, UNK: Unknown, Y/N: Yes/No.

a First treatment with pegvaliase was in the clinical trial. However, the initial treatment detailed here is post-approval, non-clinical trial. See narrative for additional information.

b Duration of initial treatment includes receiving pegvaliase during Phase 3 trial for ~3 years, discontinuing for planned pregnancy (exact stop/start dates unknown) then resuming pegvaliase post-approval for approximately 2 years before discontinuing for planned pregnancy #2.

c Individual participated in Phase 1 & 2 studies which employed weight-based dosing.

d Titration ongoing at time of advisory board presentation.

**Table 2:** Standard induction, titration and maintenance dosing approach for pegvaliase [14].

**Treatment**	**Pegvaliase dosage**	**Duration**
Induction	2.5 mg once weekly	4 weeks
Titration	2.5 mg twice weekly	1 week
	10 mg once weekly	1 week
	10 mg twice weekly	1 week
	10 mg 4 times per week	1 week
	10 mg once daily	1 week
Maintenance	20 mg once daily	24 weeks
	40 mg once daily	16 weeks
Maximum	60 mg once daily	16 weeks

#### 3.2.2 Considerations for restart when discontinuation was due to AEs (cases 1–3)

Cases 1–3 discontinued due to AEs and were off therapy for 8–61 months. Case 1 underwent a slowed titration upon restart, while cases 2 and 3 were able to restart with an expedited and standard titration schedule, respectively, and reported only mild AEs. Cases 1 and 2 achieved blood Phe ≤360 μmol/L and continued treatment, whereas case 3 ultimately discontinued after 18 months of treatment. Key considerations for restarting pegvaliase - especially following discontinuation due to an AE - included proactively addressing anxiety, optimizing premedications (eg, adding, titrating, or resuming premedications ahead of restarting pegvaliase) and ensuring access to on-demand medications to facilitate timely intervention of lower grade AEs. Furthermore, the last tolerated dose and total duration of the prior treatment course should be considered while employing a flexible approach to titration driven by individual comfort and tolerability.

#### 3.2.3 Considerations for restart when discontinuation was due to limited blood Phe response (cases 4–7)

Cases 4–7 discontinued treatment for reasons related to their blood Phe response and were off therapy for 4–64 months. Cases 4, 5, and 7 restarted therapy with an expedited titration approach and had achieved blood Phe ≤360 μmol/L at the time of presentation. AEs were mild and appeared to be less frequent compared with the first treatment course. Case 6 had only recently resumed pegvaliase therapy (2 months prior to the advisory board) and was continuing to escalate the dose with an individual-preferred slowed titration schedule, and blood Phe remained elevated. Given the longer duration of the first treatment course in these cases, the key considerations for the restart approach included the ability to resume at a higher dose and/or titrate more quickly as tolerated. However, as illustrated by case 6, a slower, collaborative approach focused on individual empowerment and communication may improve tolerability and comfort during re-initiation and titration. Additionally, an informal assessment of positive life changes (eg, increased maturity, a stable living situation, spousal support) and social determinants of health can help gauge the optimal time to restart and identify potential resources that may improve the treatment course.

#### 3.2.4 Considerations for restart when discontinuation was due to clinical trial participation (case 8)

After treatment with pegvaliase for 10 years and 3 months (with full efficacy since around 3 months post-initiation) case 8 discontinued treatment to participate in a clinical trial, remaining off pegvaliase therapy for approximately 18 months. At restart, an expedited titration schedule was well tolerated, with only mild AEs reported. Blood Phe was ≤360 μmol/L just prior to resuming therapy and within 1 month further reduced to within the physiologic range (30–120 μmol/L), allowing diet to be liberalized again. A key consideration for restarting individuals who previously experienced a sustained blood Phe response and a liberalized diet with pegvaliase is the desire to titrate more quickly if tolerated, to regain efficacy more rapidly. More frequent blood Phe monitoring may be warranted to promptly identify significant blood Phe reductions, allowing for timely dietary adjustments and dose refinement.

#### 3.2.5 Considerations for restart when discontinuation was due to pregnancy (cases 9–11)

Discontinuation for cases 9–11 were related to pregnancy, causing treatment pauses of 3–25 months. All 3 resumed pegvaliase using an expedited titration approach, which was well-tolerated, with only mild AEs reported in 2 cases. All cases achieved blood Phe ≤360 μmol/L and remained on pegvaliase therapy at the time of presentation. Key considerations for pregnancy-related discontinuations included having a balanced discussion with the individual about treatment options and ensuring collaboration between the individual, the obstetrician, and the members of the PKU care team including the genetics provider (physician or advanced practice provider) and metabolic dietitian to determine whether pegvaliase should be continued during pregnancy, depending on stage of treatment.

## 4 Discussion: key learnings of the case series

**Fig. 1:**
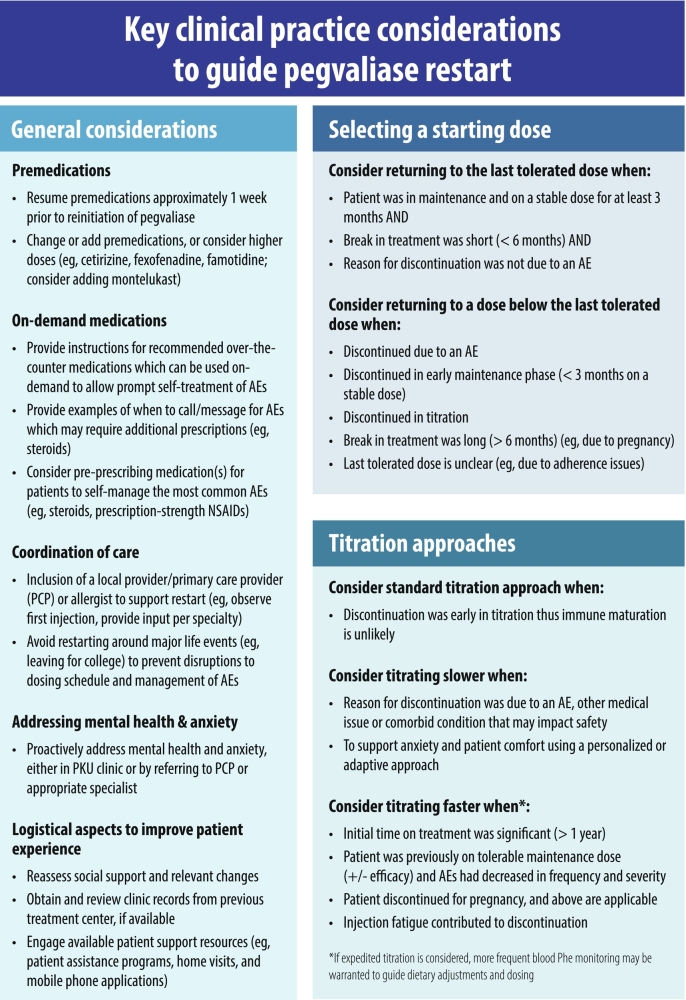
Key clinical practice considerations to guide pegvaliase restart.

Maintaining blood Phe levels within guideline-recommended targets for life is challenging [13]. Individuals with PKU should be supported in their treatment decisions for how to achieve this goal of sustained metabolic control. While individuals may choose to discontinue pegvaliase for a variety of reasons, they may ultimately wish to restart therapy to optimize their PKU management. Key considerations for pegvaliase restart are summarized in Fig. 1.

Motivations for restarting therapy included controlling blood Phe levels, improving mental clarity for school and job performance and relief from other symptoms associated with high blood Phe, such as anxiety, depression, and executive function impairments, and the ability to liberalize or normalize natural protein intake. Additionally, with factors such as increased family support and improved management of pegvaliase-related AEs, many individuals with PKU have been able to successfully resume treatment.

Restarting pegvaliase should be a shared decision-making process, acknowledging the first treatment course, individual preferences for the induction and titration approach and comfort with managing AEs. Expectation-setting remains an important component of treatment and should include a discussion of the timing of clinic or virtual visits, the desired frequency of blood Phe level monitoring and the variable time required to achieve blood Phe and dietary goals. Changes in an individual's social situation should be considered if these factors contributed to the initial treatment discontinuation. Involvement of a social worker or a clinical care coordinator may be a useful strategy to support treatment success. Discussion should be based on both positive indicators (ie, additional family support, improvements in work/life stability) and factors that may suggest timing of reinitiation is not opportune. It is important to have a conversation about the motivation to restart pegvaliase and revisit these goals throughout treatment.

The 2024 ACMG practice guidelines state they cannot recommend nor discourage the use of pegvaliase during pregnancy, not due to assumed harm but because limited data is available [2]. While there are case reports of healthy infants born to mothers continuing pegvaliase during pregnancy, there are no large, prospective studies assessing the outcomes of pegvaliase on offspring, which can contribute to the hesitancy of prescribing pegvaliase to pregnant individuals [19], [20]. The decision to continue pegvaliase treatment during pregnancy requires thoughtful consideration, bearing in mind the stage of treatment (induction/titration vs. stable maintenance phase), whether efficacy has been achieved, status of AEs, previous pregnancy outcomes (if applicable) and the likelihood of controlling blood Phe with medical nutrition therapy (with or without BH4 pharmacological agents). If it is decided to discontinue pegvaliase during or prior to pregnancy, the optimal timing for resumption should be considered carefully (ie, as soon as possible after delivery, or in the late third trimester). The goal of reinitiating pegvaliase should be to lower blood Phe levels safely and as quickly as possible to support the new parent in caring for the newborn and/or to allow for returning to an unrestricted diet [19], [20]. Limited evidence indicates that pegvaliase does not pass into breast milk and a case report showed that pegvaliase could be resumed while nursing, highlighting that the decision to breastfeed should not preclude the discussion of resuming pegvaliase [21].

There were several key learnings and takeaways from the presented cases. First and foremost, regardless of the reason for or duration of discontinuation, all individuals were able to resume treatment with pegvaliase. Proactively discussing the ability to restart pegvaliase with individuals was deemed important to ensure they understand and feel supported when making treatment decisions about managing their condition. Of note, many of the cases reported feeling worse after discontinuing their first treatment course of pegvaliase (eg, difficulties with attention, mood instability, and decreased energy), even in the absence of a significant reduction in blood Phe. There were also several important improvements during the resumption in therapy. Advisors noted that AEs were generally milder and more tolerable when compared to the first treatment course. This observation could be in part due to the immune maturation achieved over time with pegvaliase but may also extend from improved comfort with identifying and managing AEs given earlier treatment experience. It is also noteworthy that some individuals responded at lower doses or after a shorter treatment duration compared with their initial course, which may be another important observation related to the process of immune tolerization. Lastly, the learning curves experienced by both the clinician and individual during the prior treatment course with pegvaliase, including successful management strategies and familiarity with AEs, cannot be discounted and may contribute to the improved treatment experience described in many of the presented cases.

A potential limitation of the paper is that the cases discussed here do not reflect all possible clinical scenarios and individual experiences may vary. Prescription medication requiring clinician oversight should be personalized based on individual factors and optimized through ongoing clinical evaluation and follow-up. These cases and best practices are intended to share real-world experience and supplement outcomes observed with pegvaliase in the clinical trials.

## 5 Conclusion

The recently revised ACMG guidelines highlight the importance of lifelong maintenance of blood Phe levels ≤360 μmol/L for optimal neurological and intellectual outcomes in all individuals with PAH deficiency [2]. Evidence suggests that maintaining metabolic control can improve executive functioning, which may lead to improved educational and socioeconomic attainment and overall quality of life. The guidelines also strongly recommend the achievement of maternal blood Phe levels ≤360 μmol/L prior to conception and maintenance of lower blood Phe concentrations throughout pregnancy to prevent negative outcomes. Furthermore, they advise a shared decision-making approach to achieve personalized treatment goals for improving blood Phe management.

Pegvaliase can be an effective treatment option to support an individual's goals for achieving metabolic control and improving quality of life, including reducing neurocognitive sequelae and liberalizing diet. Regardless of the reason for discontinuation, resuming pegvaliase therapy after a treatment pause is feasible for most individuals with PKU and should be considered, particularly for those wanting to lower their blood Phe levels, improve dietary flexibility or optimize the management of their condition.

## CRediT authorship contribution statement

**Markey McNutt:** Writing – review & editing, Validation. **Katherine J. Anderson:** Writing – review & editing, Validation. **Ashley Andrews:** Writing – review & editing, Validation. **Erin Cooney:** Writing – review & editing, Validation. **Brittany M. Murray:** Writing – review & editing, Validation. **Michelle Tharp:** Writing – review & editing, Validation. **Erika Vucko:** Writing – review & editing, Validation. **Leah Wessenberg:** Writing – review & editing, Validation. **Kassi Wilson:** Writing – review & editing, Validation. **Derek Wong:** Writing – review & editing, Validation. **Joyanna Hansen:** Writing – review & editing, Validation, Project administration, Methodology, Conceptualization. **Sarah Rose:** Writing – review & editing, Validation, Project administration, Methodology, Conceptualization. **Bridget Wardley:** Writing – review & editing, Validation, Project administration, Methodology, Conceptualization. **Janet A. Thomas:** Writing – review & editing, Validation.

## Funding

The in-person meeting leading up to this manuscript, as well as assistance in development of the current manuscript, were funded by BioMarin Pharmaceutical Inc.

## Declaration of competing interest

The expert panel was compensated for their participation in the in-person advisory board.

Katherine J Anderson has participated in advisory boards for BioMarin, Mirum Therapeutics, and Sanofi.

Ashley Andrews has participated in advisory boards for Amgen, BioMarin, Sanofi, and Zevra Therapeutics. She participates in clinical trials as a principal investigator (PI) and sub-investigator (Sub-I) for PKU related interventions.

Erin Cooney received compensation from BioMarin for attending an advisory board related to this manuscript.

Markey McNutt has consulted and received honoraria and/or travel reimbursement from Alexion Pharmaceutical, Amgen Therapeutics, BioMarin, Chiesi, Eton Pharmaceuticals, Korro Bio, Otsuka Therapeutics, Ultragenyx, and Zevra Therapeutics. He also participated in sponsored clinical trials for Arcturus Therapeutics, BioMarin, Homology Medicines, Horizon Therapeutics, Moderna, Next Generation Gene Therapies, Otsuka Therapeutics, PTC Therapeutics, Synlogic Therapeutics, and Travere.

Brittany Murray has participated in advisory boards and consulting for BioMarin and PTC Therapeutics. She has participated in clinical trials sponsored by BioMarin.

Michelle Tharp has participated in BioMarin speaker's bureau. She has also participated in advisory boards for BioMarin, Chiesi, and Sanofi.

Janet A. Thomas has participated in advisory boards for BioMarin and PTC Therapeutics and consulting for BioMarin and Otsuka therapeutics. She has participated in clinical trials sponsored by BioMarin, Homology Medicines, 10.13039/100013223PTC therapeutics, Sanofi, and Synlogic Therapeutics.

Erika Vucko has participated in the BioMarin speaker's bureau, BioMarin consulting, and Horizon consulting. She has also participated in advisory boards for BioMarin, Horizon, and PTC. She has participated in clinical trials sponsored by BioMarin, Otsuka, and Synlogic Therapeutics.

Leah Wessenberg received compensation from BioMarin for attending an advisory board related to this manuscript. She also participated as a sub-investigator for PKU-related interventions.

Kassi Wilson received compensation from BioMarin for attending an advisory board related to this manuscript.

Derek Wong has participated in advisory boards for BioMarin and Acer Therapeutics.

Joyanna Hansen, Sarah Rose and Bridget Wardley are employees and stockholders of BioMarin.

## Data Availability

The data that has been used is confidential.

## Appendix A Supplementary data

Supplementary material Supplementary file 1: Case narratives.
