# Supplementary material for: Clinical practice considerations for restarting pegvaliase in adults with phenylketonuria
Source: Mol Genet Metab Rep. 2026 Aug 10;48:101333. doi: 10.1016/j.ymgmr.2026.101333 (PMC13486844; doi:10.1016/j.ymgmr.2026.101333)
Supplement: Supplementary file 1 [file mmc1.docx]

Supplemental material: 11 case narratives

**Case 1**

**Demographics & background**: A 45-year-old male was diagnosed with classical PKU at 17 months. Despite newborn screening (NBS), a sample error resulted in a late-diagnosis following his sister’s PKU diagnosis and concern for developmental delay. Dietary treatment with protein restriction and medical foods was initiated upon diagnosis and continued throughout adolescence and adulthood, resulting in blood Phe levels consistently between 242–484 µmol/L. He was a non-responder to sapropterin. His medical history included anxiety and depression.

**Prior course of treatment**: Being tired of following the PKU diet, he initiated treatment with pegvaliase following his sister’s positive experience and discussions with his PKU care team.

He began treatment with a baseline blood Phe level of 407 µmol/L and received cetirizine 10 mg, famotidine 10 mg, and ibuprofen 400 mg as premedications. He experienced leg cramping (not requiring treatment), heartburn (managed by increasing famotidine dose), nausea, vomiting, and vertigo (resolved following a 2-day pause in pegvaliase dosing). After ~1 month on a 20 mg daily dose, he experienced recurrent rash on both feet. In contrast to his geneticist, primary care provider, and dermatologist, he believed this was related to treatment with pegvaliase, and despite the clinic’s recommendation to continue, treatment was stopped.

**Discontinuation period & reason for restart**: The individual resumed a Phe-restricted diet with medical food. He moved and his new clinic encouraged him to reconsider treatment, reassuring him that adverse effects (AEs) would be managed adequately, and a slow titration schedule would be followed to reduce AE risk. His sister’s successful treatment with pegvaliase was an additional motivator.

**Restart course**: He resumed treatment with a blood Phe level of 672 µmol/L and premedications included higher dose cetirizine (20 mg), diphenhydramine (25 mg), and acetaminophen (500 mg) as needed. Following standard induction with 2.5 mg once weekly, titration proceeded slowly, progressing to 2.5 mg daily. Elevated bilirubin was noted on routine blood work, suspected to be related to Gilbert’s syndrome, and although the individual was reassured this was not due to pegvaliase, he stopped treatment and remained off for 3 months. At his request, he resumed therapy with a slowed titration approach (slightly faster than first restart), which was well tolerated with no further AEs. He advanced to 10 mg daily, and in response to low blood Phe levels his intact protein intake was increased and medical food protein decreased. At the time of the advisory board, he remained on pegvaliase, receiving 10 mg every other day and had received a promotion at work. This case illustrates that slow titration may reduce AEs without delaying efficacy, building trust may improve individual comfort and engagement, and setting clear expectations may reduce individual anxiety.

**Case 2**

**Demographics & background**: A 30-year-old female was diagnosed with PKU through NBS. She followed a Phe-restricted diet with medical formula until age 11, but subsequently lost metabolic control due to intolerance to the PKU diet, in part related to the flavor of the medical formula. She had 3 pregnancies, with some improvements in metabolic control during at least 1. She was identified as a modest responder to sapropterin. Her medical history included asthma, postural orthostatic tachycardia syndrome (POTS), and obesity.

**Prior course of treatment**: She was motivated to initiate treatment with pegvaliase due to the limited effectiveness of sapropterin and diet and recognition that her PKU symptoms were improved during periods of lower blood Phe levels (eg, brain fog, headaches, and fatigue).

Her baseline blood Phe level at treatment start was 1,058 µmol/L. At that time, her clinic’s protocol did not utilize premedication during induction, however, loratadine, cetirizine, famotidine, and naproxen were recommended at variable frequencies during titration in response to AEs (ISRs, rash, arthralgia, facial flushing), although her adherence was suboptimal. She also struggled with recurrent shortness of breath and wheezing, which was more likely associated with her asthma than with pegvaliase, as temporary treatment suspension lead to worsened respiratory issues. After restart once her asthma symptoms improved, her blood Phe levels dropped on a 20 mg daily dose, but she had a suspected anaphylactic episode, leading to treatment discontinuation for 7 weeks. She resumed at 2.5 mg with a local allergist, but continued to experience intermittent AEs (rash, dizziness, wheezing). After several months on 10 mg/day, treatment was discontinued again due to persistent AEs, limited therapeutic efficacy, symptomatic COVID-19, and anxiety over lack of support and childcare responsibilities.

**Discontinuation period & reason for restart**: After more than a year off therapy, she returned to sapropterin, which was ineffective, while consuming an unrestricted diet, as restricting Phe intake was too difficult to manage while caring for her children. Once her life circumstances became more stable with a new spouse providing additional support, making her less anxious about potential AEs, she was motivated to restart pegvaliase to relieve the symptoms she experienced with high blood Phe levels.

**Restart course**: Under the supervision of a local allergist, she re-initiated treatment with pegvaliase along with premedications (cetirizine 10 mg and famotidine 20 mg twice daily). At that time, her blood Phe level was 1,118 µmol/L. Very few AEs were reported (1 episode of hives and hand swelling occurring early after restarting that resolved with diphenhydramine). An expedited titration schedule was well tolerated, progressing rapidly from 2.5 mg/day for 3 days to 5 mg/day for 3 days, then 10 mg/day for 3 days, and finally to 20 mg/day. Thereafter, significant blood Phe reduction was achieved and blood Phe remained well-controlled in the physiologic range on a daily 20 mg dose at the time of the advisory board.

**Case 3**

**Demographics & background**: A 50-year-old female was diagnosed with classical PKU through NBS. She had a complex medical history with many chronic complaints including obesity and several other non-PKU-related issues. In addition, she had a history of anger, depression, anxiety, and difficulties with attention and focus, as well as 2 terminated pregnancies. She followed a Phe-restricted diet that was discontinued after the age of 10, in line with the standard of care at the time. She was non-responsive to sapropterin.

**Prior course of treatment**: Her decision to start pegvaliase was driven by a desire for improved blood Phe control and enhanced overall health.

She started pegvaliase within a clinical trial setting, with a baseline blood Phe level of 1,614 µmol/L. Premedications included loratadine 10 mg, ibuprofen 400 mg, and ranitidine 300 mg. She experienced significant injection site reactions and arthralgias, resulting in a modified titration schedule, dose reduction, and administration of diphenhydramine, ibuprofen, and cool compresses for symptom management. At 40 mg daily, she experienced 2 episodes of acute onset abdominal pain directly following pegvaliase injections, for which she visited the emergency department. She eventually discontinued due to persistent AEs.

**Discontinuation period & reason for restart**: She remained off treatment for 5 years, with an unrestricted diet and an average blood Phe level of 1,476 µmol/L. She was motivated to restart pegvaliase treatment as she desired to receive therapy that could support her overall health and wellbeing. However, treatment options were limited due to insurance constraints and unresponsiveness to cofactor therapy.

**Restart course**: At the time of restart, her baseline blood Phe level was 1,584 µmol/L. Pegvaliase was restarted using a standard titration scheme, and cetirizine 20 mg was prescribed as premedication. She had only mild injection site reactions. Because of her prior AEs with higher doses, the dosing was alternated between 20 mg and 40 mg for 6 weeks, with injections spaced 30–60 min apart to increase comfort. After advancing to a daily 40 mg dose, she encountered health concerns and social issues and reported inconsistent dosing. Ultimately, due to lack of insurance coverage and a perceived lack of interest or support from the clinic staff, she discontinued pegvaliase therapy.

**Case 4**

**Demographics & background**: A 24-year-old male was diagnosed with PKU through NBS. He was in generally good health and managed his PKU with low-Phe diet and medical food. In late adolescence, his protein intake increased substantially.

**Prior course of treatment**: He wanted to start pegvaliase treatment to be able to participate in college sports, exercise, and increase protein intake.

He started his first course of pegvaliase treatment with a baseline blood Phe level of 875 µmol/L and was prescribed cetirizine 10 mg as premedication. During treatment, he experienced injection site reactions, managed with daily cetirizine 10 mg, topical hydrocortisone, and a slowed dose titration for 6 weeks. Additional AEs included facial swelling and an episode of anaphylaxis at 20 mg daily, which was managed with cetirizine, ranitidine, and a dose reduction to 10 mg. He also experienced severe joint pain, treated with steroids and NSAIDs, which led to a further delay in dose escalation. During treatment, he had a perirectal abscess with normal colonoscopy and was diagnosed with a mild variant of Crohn’s disease. After 17 months dosing at 60 mg daily he experienced injection fatigue and only transient reduction in blood Phe (levels increased to >1,200 µmol/L with a large increase in protein intake), he discontinued for 4 months and returned to dietary treatment.

**Discontinuation period & reason for restart**: Although returning to a restricted diet with medical formula, the individual experienced difficulties with attention and focus (feeling worse at school and not thinking well). Upon hearing positive experiences of other individuals with PKU who responded to pegvaliase after more than a year of therapy, he was motivated to try pegvaliase again.

**Restart course**: At the time of restart, the individual’s baseline blood Phe level was 866 µmol/L. Pegvaliase was resumed using an expedited titration approach, starting at 10 mg once weekly and achieving 20 mg daily within 5 weeks. Early AEs (hives) resolved with prednisone, and thereafter, premedications were initiated with cetirizine 10 mg and famotidine 10 mg. He stopped his previously very high daily protein intake and attempted to be more adherent with blood Phe monitoring upon learning regular monitoring was required to continue therapy. At 60 mg daily, blood Phe levels eventually dropped into and below the normal range, and overall communication improved. At the time of the advisory board, he remained on pegvaliase, receiving a 60 mg daily dose.

**Case 5**

**Demographics & background**: A 25-year-old male was diagnosed with PKU through NBS. His Phe-restricted diet was initiated at 14 days of age, however, from early in life there were difficulties with dietary compliance and inconsistent blood Phe monitoring. His blood Phe levels fluctuated and worsened during adolescence. He had a difficult relationship with his parents, lived alone from 18 years onwards in a financially unstable situation and denied having symptoms of high blood Phe levels until early adulthood. He was not responsive to sapropterin.

**Prior course of treatment**: Pegvaliase treatment was started due to persistent difficulties with concentrating and mood lability associated with elevated blood Phe levels, as well as ongoing challenges with maintaining a low-Phe diet and consistently consuming medical formula.

His first course of pegvaliase started with a baseline blood Phe level of 1,378 µmol/L. Premedications included cetirizine 10 mg, ranitidine 150 mg, and ibuprofen 600 mg. During titration, he experienced joint pain, hives, chills (managed with diphenhydramine and ibuprofen), nausea, and injection site reactions; all resolved prior to reaching a dose of 10 mg twice weekly. After 21 months of therapy, of which 6 months were on 60 mg/day, pegvaliase was discontinued due to challenges with continuing injections with only moderate blood Phe level reduction.

**Discontinuation period & reason for restart**: During a 2.5-year period off treatment, the individual visited the clinic regularly to discuss his weight management and PKU treatment goals. He was interested in participating in a gene therapy trial but did not meet the weight eligibility criteria. He expressed motivation to restart pegvaliase therapy due to his inability to consistently consume medical formula, the high costs of low-protein foods, and allergic reactions (possible eosinophilic esophagitis) to certain raw fruits and vegetables. Additionally, he reported worsening mood lability, increased anxiety, and decreased energy.

**Restart course**: He initiated his second course of pegvaliase with a baseline blood Phe level of 1,526 µmol/L. Pegvaliase was resumed with an accelerated titration schedule, starting with 2.5 mg once weekly, followed by 2.5 mg twice weekly, before escalating directly to 10 mg daily. Dosing increased from 20 to 40 mg/day after 14 weeks. Daily premedications included famotidine 20 mg, fexofenadine 60 mg, and ibuprofen 800 mg. Compared to his first course, he experienced considerably fewer AEs, reporting only mild joint pain. Blood Phe levels started to decrease substantially on 40 mg daily and subsequently dropped to <2 µmol/L shortly after escalating to 60 mg daily. At the time of the advisory board, he remained on pegvaliase, receiving a 60 mg daily dose, awaiting subsequent blood Phe levels for further pegvaliase adjustment.

**Case 6**

**Demographics & background**: A 40-year-old female was diagnosed with classical PKU through NBS. Medical history included obesity, foot fracture, 2 pregnancies - both resulting in cesarean sections - and status post partial hysterectomy. She historically followed a Phe-restricted diet for PKU management and was noted to be a minimal responder to sapropterin dihydrochloride.

**Prior course of treatment**: She was motivated to start pegvaliase to improve control of her PKU; initial pegvaliase exposure was in the clinical trial setting but she discontinued due to a family emergency.

She began a second course of treatment with pegvaliase in the clinical setting (post-approval) at a former clinic with a baseline blood Phe of 1,155 µmol/L. Daily premedications included cetirizine 10 mg, famotidine 20 mg, and diphenhydramine 25 mg. AEs included mild injection site reactions that did not require intervention early in treatment, and a later large injection site reaction that was treated with a course of steroids. After several months of treatment with pegvaliase 20 mg daily, the individual was hesitant to increase to a 40 mg dose. She discontinued pegvaliase due to symptomatic COVID-19, reluctance to increase dose, and lack of significant blood Phe lowering.

**Discontinuation period & reason for restart**: The individual returned to a Phe-restricted diet and medical formula. She underwent weight loss surgery (sleeve gastrectomy) with hernia repair and after losing 60 pounds, she was interested in restarting pegvaliase to further support her weight loss goals. She transferred care to a new PKU clinic and vocalized a strong desire for a personalized titration schedule based on AEs and her comfort level.

**Restart course**: An individual worksheet was employed to assess discomfort, including pain and anxiety (using a visual analog scale (VAS) of 1–10), with the intention of making only 1 dosage change each week if her discomfort level was 3 or lower. Additionally, an individualized action plan was provided to assist in the management of potential AEs. She was pre-prescribed as-needed medications in advance to effectively manage potential AEs, including a course of steroids and prescription-strength NSAIDs. She had extensive education on when/how to use these medications. Daily premedications were started 2 weeks prior to resuming pegvaliase (loratadine 10 mg and famotidine 40 mg). After a treatment pause of 30 months, pegvaliase was resumed at 2.5 mg once weekly for 4 weeks. Utilizing the VAS to guide titration, she had advanced to 10 mg twice weekly by week 10 of treatment; AEs were noted as mild injection-site reactions, redness, and itching that resolved without treatment. At the time of the advisory board, titration was ongoing with a planned dose increase to 10 mg, 4x/weekly.

**Case 7**

**Demographics & background**: A 37-year-old male was diagnosed with PKU through NBS. His medical history included depression, short-term memory problems, ADHD, anxiety and irritability, a disc fracture, back and knee aches, balance problems, headaches, and vertigo. He had been managed on a Phe-restricted diet but began struggling with adherence during adolescence. He was off diet for the majority of his adult years.

**Prior course of treatment**: He was motivated to start pegvaliase therapy due to experiencing negative effects of persistently elevated blood Phe levels, a longstanding difficulty adhering to a Phe-restricted diet (especially avoiding high-protein foods), and an interest in participating in a clinical trial.

He began his first pegvaliase course in a clinical trial setting with a baseline blood Phe level of 2,052 µmol/L, while not restricting Phe intake but consuming some medical formula. No premedications were prescribed, and no AEs reported. During the trial, the individual repeatedly missed clinic visits and dosing despite multiple contact attempts by the clinical team, leading to withdrawal from the study.

**Discontinuation period & reason for restart**: As the indidivual experienced negative effects of high blood Phe levels, he attempted to reduce his intake of high-protein foods and tried consuming medical formula. He became lost to follow-up and remained off pegvaliase for 8 years. Over time, he had neuropsychiatric symptoms including anxiety (requiring medication), attention deficit disorder, irritability, and depression requiring hospitalization, but also impaired balance and headaches. He noticed a decline in concentration and work performance, and recalled having better memory while receiving pegvaliase. Following FDA approval of pegvaliase, he returned to the clinic requesting to restart therapy.

**Restart course**: At the time of treatment reinitiation, the individual’s baseline blood Phe level was 1,609 µmol/L. Premedications, including cetirizine 10 mg and ranitidine 150 mg, were prescribed and well-tolerated. He resumed pegvaliase with an expedited titration schedule, advancing from 2.5 mg twice weekly to 20 mg daily by week 9. Early in treatment, he experienced mild joint pain, which resolved either spontaneously or with ibuprofen. His dose was escalated to 40 mg daily at week 60 and further increased to 60 mg daily at week 120. After dose escalating to 80 mg daily, his blood Phe levels eventually dropped. To address social determinants of health that could affect adherence, the individual received assistance from the National Organization for Rare Disorders to cover clinic appointments and transportation. The partnership with social work and greater flexibility compared with the clinical trial setting allowed for improved indidivual support to comply with follow-up visits. At the time of the advisory board, he was continuing pegvaliase on an 80 mg daily dose.

**Case 8**

**Demographics & background**: A 37-year-old female was diagnosed with PKU through NBS. She adhered to a Phe-restricted diet and medical formula. She participated in a sapropterin dihydrochloride clinical trial and responded to treatment, though not enough to allow for dietary liberalization.

**Prior course of treatment**: Seeking to liberalize her diet and improve control of her blood Phe levels, she expressed interest in enrolling in a pegvaliase clinical trial.

She began her first course of pegvaliase in the trial setting with a baseline blood Phe level of 600 µmol/L. She was not prescribed any premedications and the titration schedule was unknown (although, faster than the current label). Early AEs included full body rash, hives, and severe joint pain. After 3 months on pegvaliase, her blood Phe levels dropped to around 18 µmol/L, and the AEs resolved soon after. Her diet was liberalized per the clinical trial protocol in response to hypophenylalaninemia (hypoPhe). She remained on treatment (20 mg, 3x/week) for about 10 years, maintaining blood Phe levels between 60-100 µmol/L with an unrestricted diet. However, she wished to prepare for a potential pregnancy and explore whether adequate blood Phe control could be achieved with another therapeutic option while allowing her to maintain a sufficiently liberalized diet. Moreover, she desired a break from injections and more consistent blood Phe levels (avoiding hypoPhe). To pursue these goals, she enrolled in a clinical trial for an investigational new drug (sepiapterin), which required pegvaliase discontinuation.

**Discontinuation period & reason for restart**: The individual was motivated to restart pegvaliase therapy because she could not fully liberalize her diet with sepiapterin. In addition, she aimed to achieve normal blood Phe levels again, as she felt dissatisfied when levels exceeded 120 µmol/L.

**Restart course**: At restart, her baseline blood Phe level was 276.1 µmol/L. Daily premedications included cetirizine 10 mg and famotidine 20 mg. An expedited titration approach (2.5 mg 2x/week for 1 week, 2.5 mg 4x/week for 1 week, 10 mg 2x week for 1 week) to 10 mg daily resulted in a clinical response. Compared with her first course of pegvaliase, she experienced significantly faster efficacy, fewer AEs (limited to mild hives and injection site reactions that resolved spontaneously), and greater flexibility in dosing. As her blood Phe levels remained below 30 µmol/L, intact protein intake was increased by 10-20 g/day and diet was liberalized again. However, ongoing hypoPhe despite an unrestricted diet required a decrease in pegvaliase dosing. At the time of the advisory board, she remained on pegvaliase, receiving 10 mg, 4x/week.

**Case 9**

**Demographics & background**: A 28-year-old female was diagnosed with classical PKU through NBS. She was initially managed with a Phe-restricted diet and medical formula but discontinued at age 14. However, at the age of 23, she returned to clinic and made efforts to resume a Phe-restricted diet by selecting lower-protein foods, limiting meat intake, and starting medical formula.

**Prior course of treatment**: Her motivation to initiate pegvaliase was a desire to become pregnant.

She began her first pegvaliase course with a baseline blood Phe level of 1,440 µmol/L. Premedications included cetirizine 10 mg and famotidine 20 mg. She experienced mild AEs, including headache, injection site reactions, joint pain and indigestion, which either resolved spontaneously or were managed with warm baths, ibuprofen, and hydrocortisone cream. She had reduced blood Phe levels to within guideline-recommended ranges and after ~75 weeks on pegvaliase, she became pregnant. Approximately 2 months after delivering a healthy child, she decided to discontinue pegvaliase due to difficulties remembering daily injections while caring for her newborn.

**Discontinuation period & reason for restart**: The individual was not on any dietary treatment during the 3-month break and experienced symptoms of anxiety and depression, that she felt were more severe than when blood Phe levels were better controlled. In addition, she found it difficult to care for her infant. She decided to restart pegvaliase, as she also wanted to optimize her PKU management in case she would want to have more children in the future.

**Restart course**: At the time of restart, her baseline blood Phe level was 1,357 µmol/L. Premedications included cetirizine 10 mg and famotidine 20 mg. An expedited titration approach was implemented, starting with 20 mg 5x/week. She had rare injection site redness, headache and joint pain that resolved with a warm bath and ibuprofen. Three months after restarting, she became lost to follow-up, and no blood Phe levels were provided for 9 months. During that time, she restarted herself at 20 mg 3x/week. After reconnecting with the clinic, the dosing frequency was increased to 20 mg daily, blood Phe levels were monitored more regularly, and injections were administered more consistently. Despite these efforts, dosing often remained inconsistent, resulting in fluctuating blood Phe levels. At the time of the advisory board, she was continuing pegvaliase 30 mg daily for 2 days, alternating with 20 mg daily for 1 day.

**Case 10**

**Demographics & background**: A 42-year-old female was diagnosed with PKU through NBS. She initially followed a Phe-restricted diet and consumed medical food, but discontinued during adolescence, leading to chronic high blood Phe levels. She was identified as a non-responder to sapropterin. Her medical history included anxiety with panic attacks. Later in life, she resumed diet and eventually restarted drinking medical formula. However, she continued to struggle managing daily tasks and work, and experienced persistent anxiety, depression, and brain fog.

**Prior course of treatment**: Motivated by a desire to control her blood Phe levels to improve her brain fog and to plan for pregnancy, she enrolled in a pegvaliase clinical trial.

She began her first pegvaliase course as part of a clinical trial, with a baseline blood Phe level of 1,200 µmol/L. Reported AEs included injection site reactions, lymphadenopathy, joint pain, fever, hives, and lip swelling (managed with diphenhydramine and a pause in injections). Subsequently, cetirizine and famotidine were prescribed as premedications. She responded to treatment, but pegvaliase was discontinued due to a planned pregnancy. Following childbirth, she resumed pegvaliase while breastfeeding but discontinued again to re-establish dietary control in preparation for a second pregnancy.

**Discontinuation period & reason for restart**: While her blood Phe levels remained within treatment range with a protein-restricted diet and medical formula, she began experiencing symptoms similar to those present prior to treatment, including brain fog, mental health issues, and impaired daily functioning. Desiring improved quality of life and greater dietary flexibility, she elected to restart pegvaliase therapy.

**Restart course**: Prior to restarting pegvaliase, her baseline blood Phe level was 868 µmol/L. An accelerated titration regimen was employed, starting with 10 mg 4x/week for 1 week and progressing over 5 weeks to a maintenance dose of 40 mg daily. She had fewer AEs than before, reporting only intermittent localized hives, and achieved efficacy faster. She was lost to clinic follow-up for approximately 3 months and struggled with treatment adherence. At the time of the advisory board, she had 3 recent blood Phe levels, all in the target range, with a 60 mg daily dose and an unrestricted diet.

**Case 11**

**Demographics & background**: A 35-year-old female was diagnosed with PKU through NBS. Dietary treatment was initiated at 2 weeks of age, but maintaining stable blood Phe levels within the target range was difficult throughout life. Limited parental support and financial stressors related to maintaining a low-Phe diet further complicated management.

**Prior course of treatment**: She was motivated to start pegvaliase to improve dietary flexibility, allowing her to eat the same foods as her family, and to gain mental clarity as she navigated nursing school.

She began her first course of pegvaliase with a baseline blood Phe level of 1,636 µmol/L. Daily premedications included loratadine 10 mg and ranitidine 75 mg. Following a drop in blood Phe levels on a 20 mg daily dose, she developed alopecia, which resolved with alternating 10 mg and 20 mg doses. In response to the low blood Phe levels, supplements including multivitamin, biotin, B_12_, vitamin D, and zinc were administered, and she was encouraged to increase her intact protein intake. Pegvaliase therapy was eventually discontinued due to a planned pregnancy.

**Discontinuation period & reason for restart**: During the off-treatment period, the individual increased her medical formula intake, but her blood Phe levels immediately exceeded the target range, with an average level of 870 µmol/L during pregnancy, and her child was affected by maternal PKU. After childbirth and breastfeeding, she decided to restart pegvaliase treatment due to persistently high blood Phe levels (>1,400 µmol/L) and impaired cognitive clarity during her studies.

**Restart course**: At the time of restart, her baseline blood Phe level was 1,474 µmol/L. She was prescribed loratadine 10 mg as premedication and following an expedited titration schedule (starting with 2.5 mg 2x/week before increasing to 10 mg daily over the course of 4 weeks and then establishing a maintenance dose of 20 mg daily by week 6), she responded quickly to daily injections. At approximately 36 weeks, on 20 mg daily, she reported night sweats, leading to a dose adjustment to 20 mg every other day, which resolved the symptoms. As her blood Phe levels increased again, she resumed daily dosing, alternating between 10 and 20 mg, which resulted in a decline in blood Phe levels into the physiologic range. Eventually, she chose to continue treatment for another pregnancy, during which her blood Phe levels have remained within the physiologic range.
